# Characteristics of positive horizontal margins in patients who underwent colorectal endoscopic submucosal dissection

**DOI:** 10.1002/deo2.300

**Published:** 2023-10-12

**Authors:** Kazumasa Kawashima, Takuto Hikichi, Michio Onizawa, Naohiko Gunji, Yu Watahiki, Chiharu Sakuma, Tomoaki Mochimaru, Mai Murakami, Osamu Suzuki, Yuko Hashimoto, Masao Kobayakawa, Hiromasa Ohira

**Affiliations:** ^1^ Department of Gastroenterology Fukushima Medical University Fukushima Japan; ^2^ Department of Endoscopy Fukushima Medical University Hospital Fukushima Japan; ^3^ Department of Diagnostic Pathology Fukushima Medical University Fukushima Japan; ^4^ Medical Research Center Fukushima Medical University Fukushima Japan

**Keywords:** colorectal neoplasm, endoscopic submucosal dissection, fibrosis, horizontal margin, lesion size

## Abstract

**Objectives:**

Endoscopic submucosal dissection (ESD) enables en bloc resection of colorectal neoplasms, but occasionally results in positive horizontal margins (HMs). However, the site of the resected specimen that tends to be positive for HM has not been investigated. We aimed to clarify the characteristics associated with HMs in lesions resected en bloc with ESD.

**Methods:**

Patients with colorectal neoplasms who underwent en bloc resection with ESD were included in this study. The patients were divided into negative HMs (HM0) and positive or indeterminate HMs (HM1) groups. The characteristics associated with HM1 resection were investigated. In addition, the local recurrence rate during endoscopic follow‐up for >6 months after ESD was observed.

**Results:**

In total, 201 lesions were analyzed in 189 patients (HM0, 189 lesions; HM1, 12 lesions). The HM1 group had a significantly larger median lesion diameter (25 vs. 55 mm; *p* < 0.001) and more lesions with >50% circumference than did the HM0 group (*p* < 0.001). Furthermore, the prevalence of severe fibrosis was significantly higher in the HM1 group than in the HM0 group (*p* < 0.001). Positive horizontal sites of the resected specimens were more frequent at the oral and anal sites than at the lateral sites. No local recurrences were observed in either group.

**Conclusions:**

The characteristics associated with HM1 depended on lesion size, particularly lesions with >50% circumference, and submucosal fibrosis.

## INTRODUCTION

Endoscopic submucosal dissection (ESD) enables en bloc resection regardless of lesion size and allows for precise pathological diagnosis.^1^, ^2^, ^3^, ^4^, ^5^ Criteria for curative resection after endoscopic resection include histological findings, such as negative vertical margins (VMs), submucosal (SM) invasion <1000 μm from the muscularis mucosae, histological types (papillary adenocarcinoma, tubular adenocarcinoma, and medullary carcinoma), budding grade, and negative lymphovascular invasions (LVIs).^6^ When these conditions are not met, surgery is recommended due to the possibility of regional lymph node metastasis. When the horizontal margin (HM) is positive or indeterminate without these risk factors for lymph node metastasis, it is also considered non‐curative resection. Piecemeal resection using endoscopic resection is associated with a significantly higher local recurrence rate than en bloc resection.^7^, ^8^, ^9^, ^10^, ^11^, ^12^ Recent improvements in endoscopic devices and various endoscopic treatment methods have reduced the outcomes of non‐curative resection.^13^, ^14^, ^15^, ^16^, ^17^, ^18^ However, even after en bloc resection with ESD, the 5‐year local recurrence rate in patients with a positive HM was as high as 2.1%–40%.^19^, ^20^, ^21^ The characteristics and local recurrence associated with positive HMs in en bloc resection of colorectal neoplasms using ESD remain unclear. Additionally, to the best of our knowledge, no study has investigated the site of the resected specimen that tends to be positive for HM. Herein, we aimed to investigate the characteristics and sites associated with positive HM in colorectal neoplasms that were resected en bloc with ESD.

## METHODS

### Study design

We conducted a single‐center, retrospective cohort study. Patients who underwent colorectal ESD at Fukushima Medical University Hospital between January 2017 and December 2021 were included. Patients with nonepithelial lesions or those who underwent ESD with an attempt at en bloc resection that resulted in piecemeal resection were excluded. Furthermore, among patients with an endoscopic follow‐up of >6 months after ESD, local recurrence was observed. Patient characteristics and lesions were extracted from the electronic medical records. Lesions with negative HMs were defined as the HM0 group, whereas those with positive or indeterminate HMs were defined as the HM1 group.

Macroscopic types were classified as protruding tumors (Is) or laterally spreading tumors (LST). LST was first divided into granular LST (LST‐G) and nongranular LST. Furthermore, the LST‐G was subdivided into homogeneous type and nodular mixed type, according to Kudo's classification.^22^ Informed consent for endoscopic treatment was obtained from all the patients. This study was conducted in accordance with the guidelines of the Declaration of Helsinki and approved by the Institutional Ethics Committee of Fukushima Medical University (protocol code: 2407, date of approval: April 28, 2020).

### ESD procedure

ESD was performed using a single‐channel colonoscope equipped with a water‐jet system (PCF‐Q260J or PCF‐H290TI; Olympus Optical Co., Ltd.). A disposable transparent attachment (DH‐28R; Fujifilm Medical Co., Ltd.) was attached to the tip of the colonoscope to facilitate ESD. Prior to ESD, circumferential marking was performed 5 mm outside the periphery of the lesion, using the tip of a dual knife (KD‐650Q; Olympus). Glycerol with diluted indigo carmine was sufficiently injected into the submucosa on the anal side, followed by a sodium hyaluronate solution. A mucosal incision was made on the anal side and SM dissection was performed using a dual knife and an IT knife nano (KD‐612Q; Olympus). The traction methods included a clip‐and‐threaded technique,^23^ S–O clip traction,^16^ and a multi‐loop traction device.^15^ The choice of the traction method was based on the operator's discretion. An electrosurgical generator (VIO 300D or VIO 3; Erbe, Elektromedizin GmbH) was used for circumferential incision and SM dissection. No residual lesions or markings were immediately observed around the margin of the ulcer just after ESD.

### Histological evaluation of ESD specimen

The resected specimens were immersed in 4% formalin for 24–48 h. The specimens were embedded in 10% paraffin, cut into 2‐mm slices, and stained with hematoxylin and eosin (H&E staining). Histological findings were classified according to the Japanese Classification of Colorectal, Appendiceal, and Anal Carcinom,^6^ and all specimens were examined by two experienced pathologists. The HM status and the lesions closest to the outer periphery side were carefully determined and we made sections from the outside to the inside. Furthermore, the HM1 cases were reviewed by an experienced pathologist. To determine the anal side of the lesion, we placed a double marking on the anal side outside the lesion during ESD and compared the preoperative lesion shape with the resected specimen. When the diagnosis of LVI was inconclusive, Elastica van Gieson staining for vascular invasion and antibody D2‐40 staining for lymphatic invasion were performed. En bloc resection was defined as resection of a lesion in one piece without fragmentation, and R0 resection was defined as en bloc resection with tumor‐free HM and VM. The areas of the resected specimen and lesions (mm^2^) were calculated based on a previous report.^24^ Invasion depth was classified as follows: Tis, mucosal carcinoma; T1a, a lesion extending up to 1000 μm below the muscularis mucosa; and T1b, a lesion more than 1000 μm from the muscularis mucosa.

### Follow‐up after ESD

Post ESD, a magnified colonoscopy with indigo carmine was performed every 12 months for curative resection and every 6 months for noncurative resection.^25^ Local recurrence was diagnosed when a tumor was detected at an ESD scar and tumor cells were histologically verified using a biopsy specimen.

### Outcomes

ESD outcomes, including operators, histological types, LVIs, areas of the resected specimen and lesion, degree of fibrosis, and sites of positive HM in HM1 resections, were investigated. Moreover, the marking and the procedure time, en bloc and R0 resection rates, adverse events (AEs), and local recurrence rates were evaluated.

SM fibrosis was classified as F0 (none), F1 (mild), and F2 (severe) according to a previous report.^26^ Marking time was defined as the time from the start of marking to the start of mucosal incision, and procedure time was defined as the time from mucosal incision to the completion of SM dissection. Operators with more than 40 cases of experience in colorectal ESD were defined as experts.^27^

AEs included intraoperative perforation, postoperative bleeding, and postoperative stenosis. Intraoperative perforation was defined as the confirmation of a muscle layer defect and intra‐abdominal fat endoscopically. Postoperative bleeding was defined as requiring blood transfusion, endoscopic hemostasis after endoscopic treatment, or a decreased hemoglobin level >2 g/dL. Postoperative stenosis was defined as endoscopic balloon dilation.

### Statistical analysis

Categorical variables were compared using the chi‐square test or Fisher's exact test, and quantitative variables were analyzed using the Mann–Whitney *U* test. Statistical significance was set at *p* < 0.05. Cumulative local recurrence was measured from the date of endoscopic resection to the date of local recurrence from ESD. All statistical analyses were performed using EZR (Saitama Medical Center, Jichi Medical University), a graphical user interface for R (The R Foundation for Statistical Computing).^28^ More precisely, it is a modified version of the R commander designed to add statistical functions frequently used in biostatistics.

## RESULTS

### Patient characteristics

**FIGURE 1:**
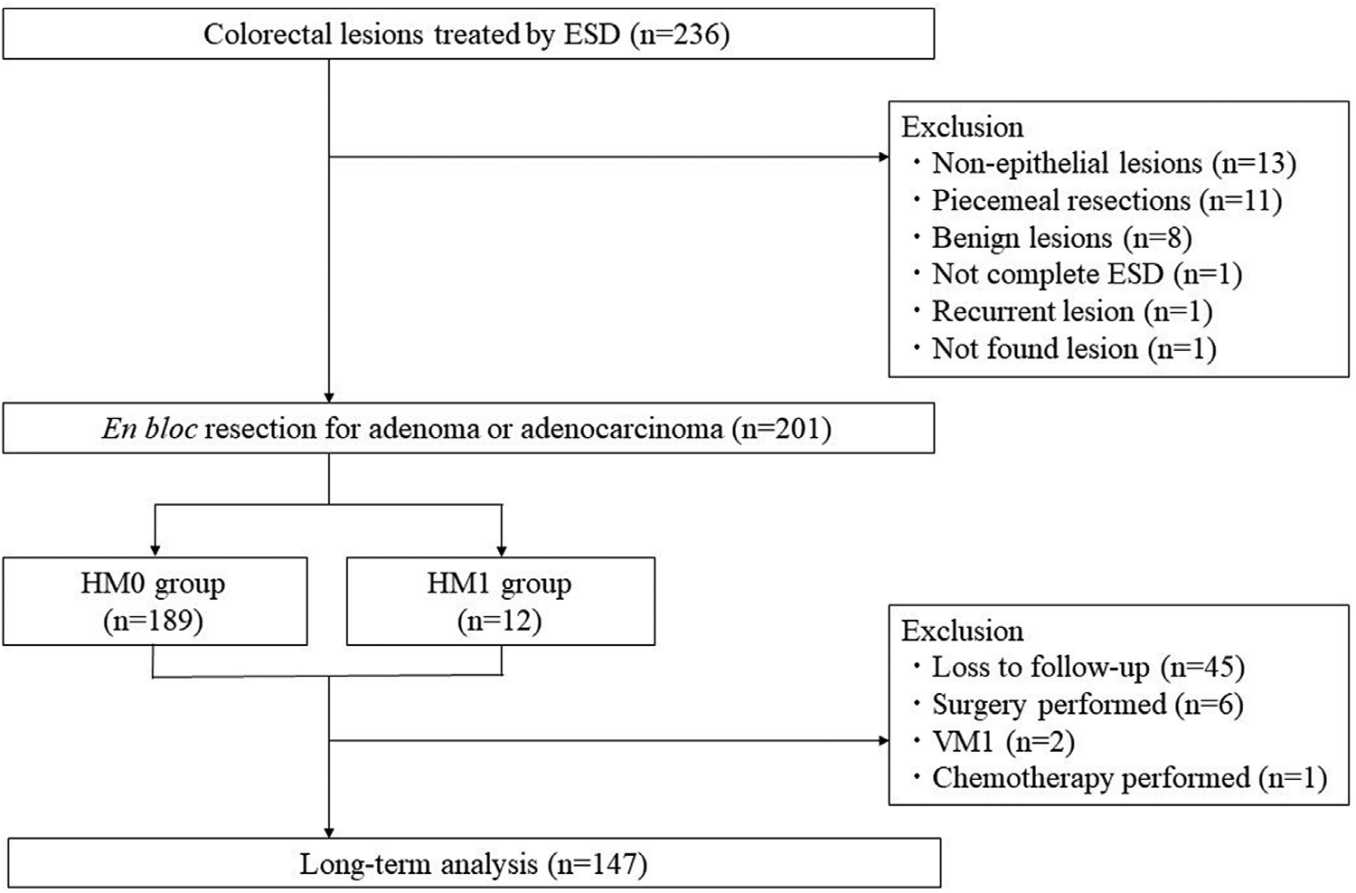
Flowchart of the study. ESD, endoscopic submucosal dissection; HM0, lesions with negative horizontal margins; HM1, lesions with positive or indeterminate horizontal margins; VM1, lesions with positive vertical margins.

Among 236 lesions in 221 patients treated with ESD, 201 lesions in 189 patients pathologically diagnosed with adenoma or adenocarcinoma up to SM invasion were included (Figure 1). For the long‐term analysis, 147 lesions in 147 patients who underwent endoscopic follow‐up for more than 6 months after ESD were retrieved. There were no significant differences in patient characteristics between both groups (Table 1).

**TABLE 1:** Patient and lesion characteristics at baseline before colorectal endoscopic submucosal dissection (*n* = 201).

	HM0 group	HM1 group	*p*‐value
Patients, *n*	189	12	−
Age, years, median (range)	73 (46–92)	67 (48–78)	0.132
Gender (male/female)	112/77	7/5	0.950
Antithrombotic drugs, *n* (%)	27 (14.3)	0	0.129
Location, *n*			
Right colon/left colon/rectum	88/32/69	5/3/4	0.774
Macroscopic type, *n*			
Is/LST‐G/LST‐NG	15/131/43	0/11/1	0.244
Lesion diameter (mm), median (range)	25 (5–123)	55 (30–93)	<0.001
Circumferential range of the lesion, *n*			
<50%/≥50%	171/18	5/7	<0.001

*Notes*: The right colon includes the cecum, ascending colon, and transverse colon. The left colon includes the descending and sigmoid colon.

Abbreviations: HM0, lesions with negative horizontal margins; HM1, lesions with positive or indeterminate horizontal margins; LST‐G, granular type of laterally spreading lesion; LST‐NG, nongranular type of laterally spreading lesion.

### Characteristics associated with HM1 resections

The clinicopathological features of the 201 lesions, including 189 lesions treated with HM0 resection and 12 lesions treated with HM1 resection, are shown in Table 2. There were no significant differences in lesion locations, macroscopic types, traction, or single balloon‐over tube usage, marking time or histological types, including LVIs, between the HM0 and HM1 groups. The HM1 group had a significantly larger median lesion diameter (25 vs. 55 mm; *p* < 0.001) and significantly more lesions with >50% circumference than did the HM0 group (*p* < 0.001). The median resected lesion and specimen areas were significantly larger in the HM1 group (3.5 vs. 17.3 cm^2^; *p* < 0.001 and 9.9 vs. 23.6 cm^2^; *p* < 0.001, respectively). The prevalence of F2 fibrosis in the HM1 group was significantly higher than that in the HM0 group (*p* < 0.001). The median procedure times were 80 min in the HM0 group and 238 min in the HM1 group, with R0 resection rates of 97.9% and 0%, respectively.

**TABLE 2:** Outcomes of colorectal endoscopic submucosal dissection.

	HM0 group	HM1 group	*p*‐value
Operators, *n*			
Expert/non‐expert	147/42	7/5	0.123
Tractions, *n* (%)	100 (52.9)	8 (66.7)	0.354
Single balloon‐over tube, *n* (%)	65 (73.9)	3 (60.0)	0.608
Marking time (min), median (range)	9 (2−29)	9 (7−17)	0.299
Procedure time (min), median (range)	80 (10−338)	238 (88−600)	<0.001
R0 resection, *n* (%)	185 (97.9)	0	<0.001
Histological types, *n*			
Adenoma/Tis/T1a/T1b	87/74/11/17	2/7/0/3	0.077
Lymphatic invasion, *n* (%)	7 (3.7)	0	0.497
Vascular invasion, *n* (%)	9 (4.8)	0	0.439
Resected lesion area (cm^2^), median (range)	3.5 (0.1–97)	17.3 (6–51)	<0.001
Resected specimen area (cm^2^), median (range)	9.9 (1–141)	23.6 (7–64)	<0.001
Fibrosis, *n*			
F0 + F1/F2	178/11	8/4	<0.001

*Notes*: Single balloon‐over tube was only used for right colon lesion. Marking time was defined as the time from the start marking to the start mucosal incision, and procedure time was defined as the time from mucosal incision to the completion of submucosal dissection.

Abbreviations: F0, no fibrosis; F1, mild fibrosis; F2, severe fibrosis; HM0, lesions with negative horizontal margins; HM1, lesions with positive or indeterminate horizontal margins; R0 resection, en bloc resection with tumor‐free horizontal and vertical margin; Tis, mucosal carcinoma; T1a, lesion extending up to 1000 μm below the muscularis mucosa; T1b, lesion more than 1000 μm from the muscularis mucosa.

### Adverse events

Eight of the included patients experienced AEs. In the HM0 group, five patients had intraoperative perforations and one had postoperative bleeding. In the HM1 group, one patient had an intraoperative perforation and one had postoperative stenosis. The characteristics associated with AEs are shown in Table 3. Lesions exceeding 50% of the circumference, no traction use, and positive HMs were significantly associated with AEs; however, no association was observed with fibrosis or operators.

**TABLE 3:** Outcomes of adverse events.

	Without AE (*n* = 193)	With AE (*n* = 8)	*p* Value
Antithrombotic drugs, *n* (%)	37 (19.2)	1 (12.5)	1.000
Circumferential range of the lesion, *n*			
<50%/≥50%	171/22	5/3	0.028
Operators, *n*			
Expert/Non expert	148/45	6/2	0.912
Tractions, *n* (%)	107 (55.4)	1 (12.5)	0.017
Single balloon‐over tube, *n* (%)	62 (71.3)	6 (100)	0.186
Histological types, *n*			
Adenoma/Tis/T1a/T1b	86/79/9/19	3/2/2/1	0.092
Marking time (min), median (range)	9 (2–29)	10 (5–13)	0.918
Procedure time, min, median (range)	82 (10–660)	143 (30–358)	0.195
En bloc resection rate, *n* (%)	193 (100)	8 (100)	−
R0 resection rate, *n* (%)	179 (92.7)	6 (75.0)	0.069
HM0/HM1, *n*	183/10	6/2	0.020
Lymphatic invasion, *n* (%)	7 (3.6)	0	0.584
Vascular invasion, *n* (%)	9 (4.7)	0	0.532
Fibrosis, *n*			
F0 + F1/F2	180/13	6/2	0.054

*Notes*: Single balloon‐over tube was only used for right colon lesion. Marking time was defined as the time from the start marking to the start mucosal incision, and procedure time was defined as the time from mucosal incision to the completion of submucosal dissection.

Abbreviations: AE, adverse events; F0, no fibrosis; F1, mild fibrosis; F2, severe fibrosis; HM0, lesions with negative horizontal margin; HM1, lesions with positive or indeterminate horizontal margin; R0 resection, en bloc resection with tumor‐free horizontal and vertical margin; Tis, mucosal carcinoma; T1a, a lesion extending up to 1000 μm below the muscularis mucosa; T1b, a lesion more than 1000 μm from the muscularis mucosa.

### Long‐term follow‐up

A total of 147 patients, including 138 in the HM0 group and 9 in the HM1 group, were included in the analysis. The median follow‐up period for each group was 23 months, with no significant differences. No local recurrence was observed in either group during follow‐up.

### Sites associated with HM1 resections

**FIGURE 2:**
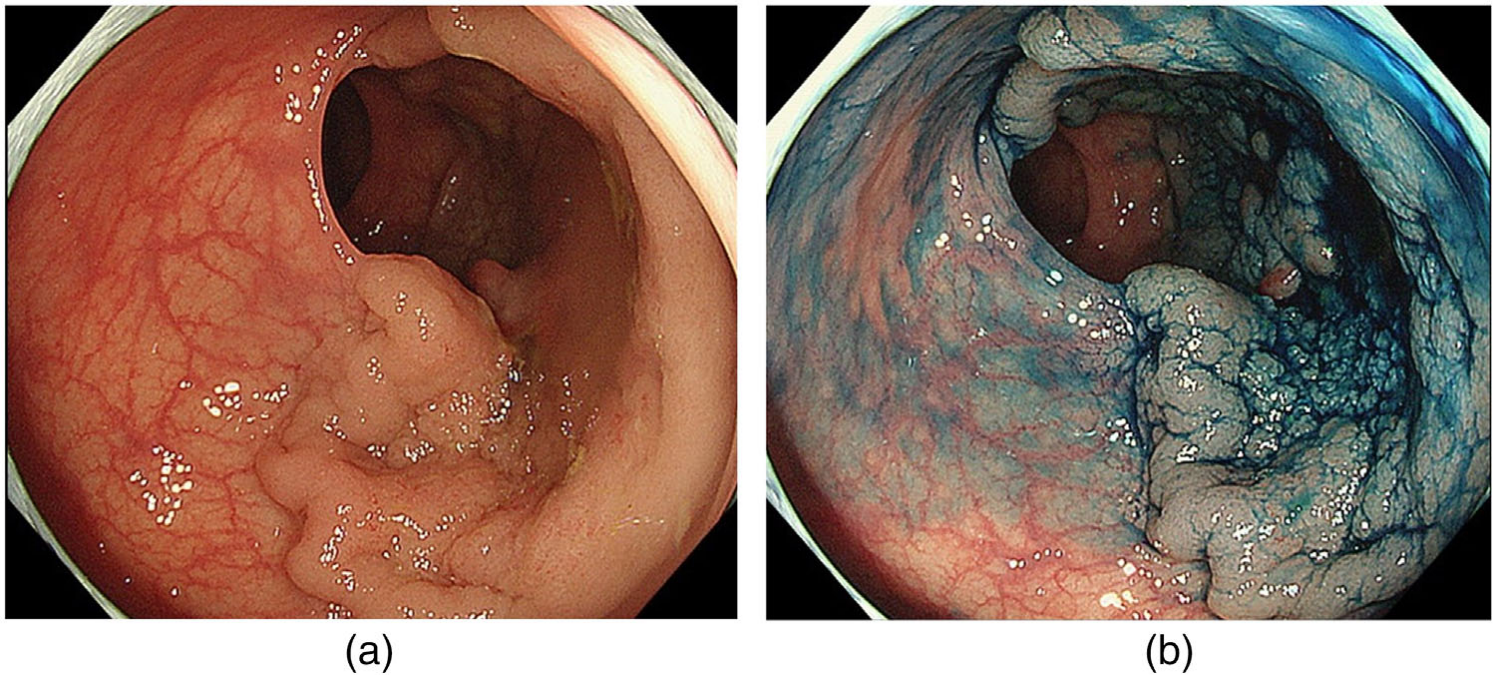
Endoscopic images in case 1 with positive horizontal margin resection. Images under white light (a) and after spraying with indigo carmine (b). The granular‐type laterally spreading lesion with a nodular mixed type in the rectosigmoid colon occupied 75% of the circumference.

**FIGURE 3:**
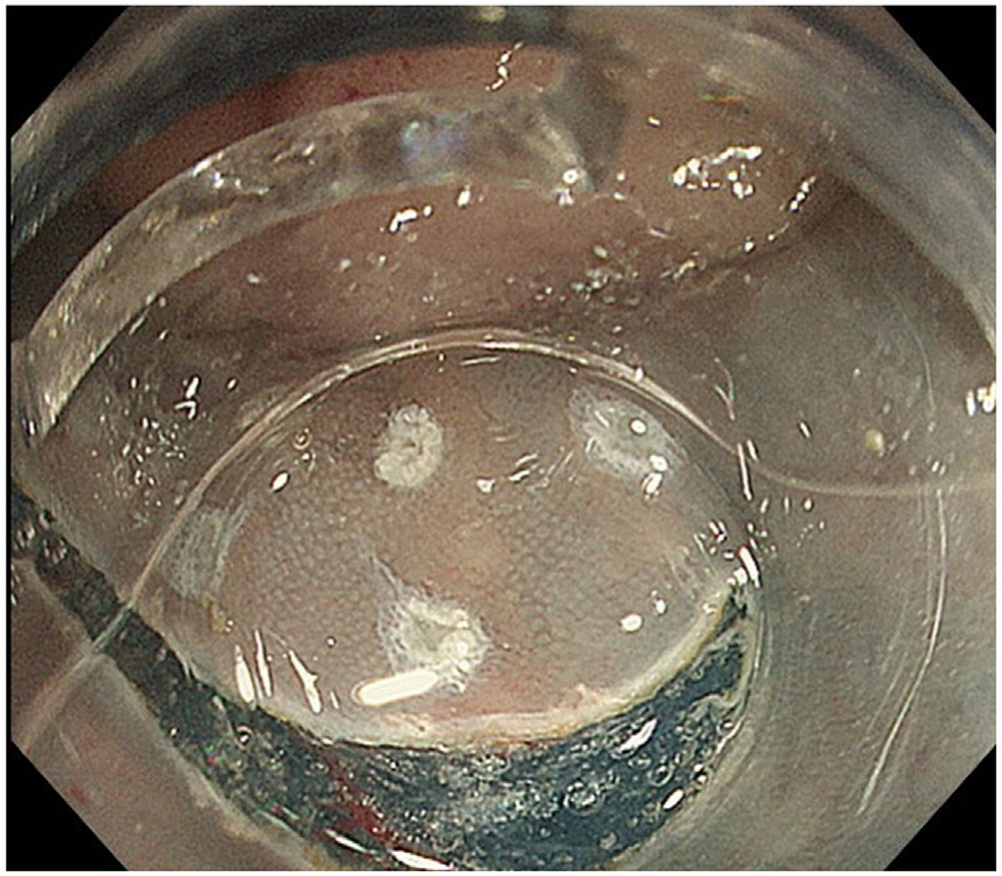
Incision line in the endoscopic submucosal dissection procedure. The incision was performed outside the lesion according to the markings.

**FIGURE 4:**
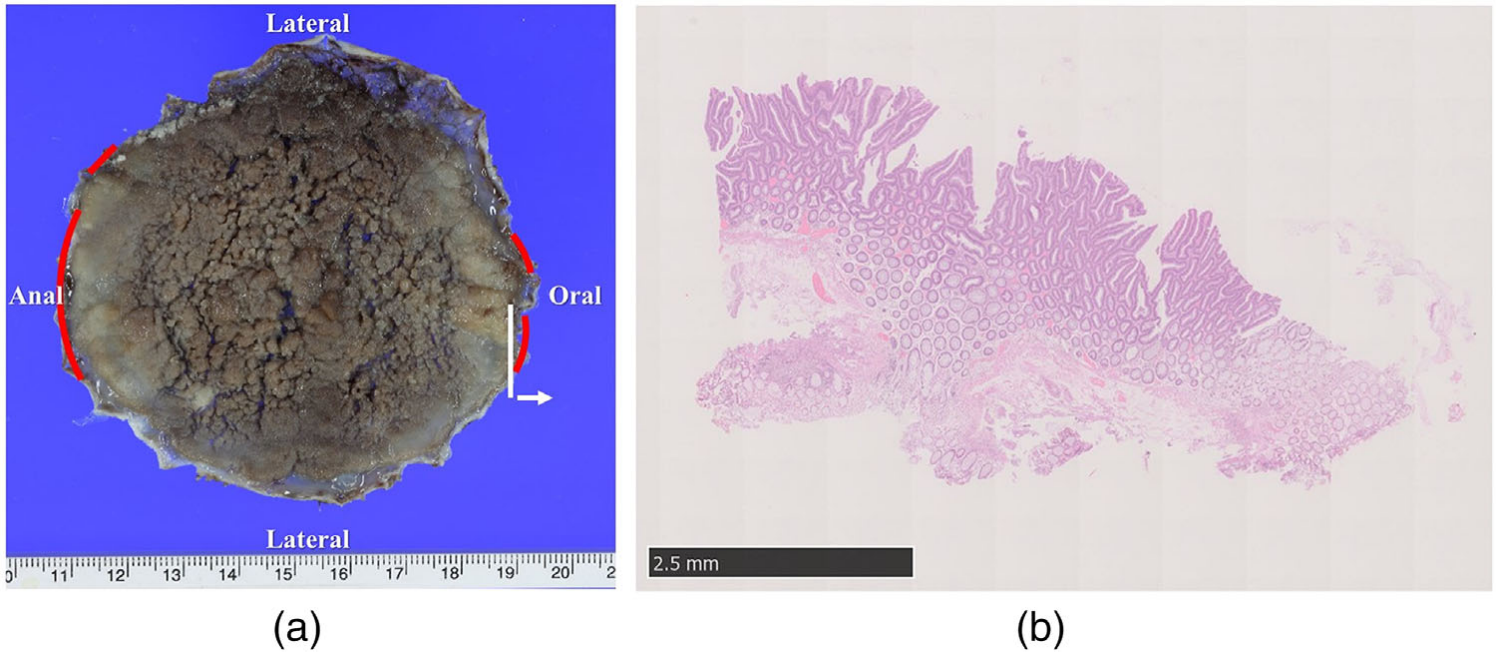
Resected specimen in case 1 with the positive horizontal margin resection (a) The size of resected specimen is 85 × 80 mm. The sites of positive horizontal margin are on both anal and oral site, indicated by redlines. White line shows the initial section of slice, and white arrow indicates the direction of diagnosing the pathology. (b) Adenoma was present on the initial section of slices on the white line in (a).

In the HM1 group, 10 adenoma lesions and 2 adenocarcinoma lesions were histopathologically diagnosed at positive HM sites. Furthermore, the positive sites of the resected specimens included eight lesions on the anal site, five lesions on the oral site, and three lesions on the lateral site, with duplicates (Table 4). As shown in Table 4, one T1b patient (case 12) with negative VMs underwent additional surgical resection, and the remaining two T1b patients (case 1: with positive VMs and case 2: with negative VMs) were followed up without additional surgical resection due old age and underlying disease (schizophrenia), respectively. After all, 11 patients were followed up after consultation. Case 1 in the HM1 group is shown in Figure 2. The incision was made outside the lesion (Figure 3); however, the histology of the positive site was an adenoma, and the positive sites were on both the oral and anal sides (Figure 4a,b).

**TABLE 4:** Clinical outcomes of patients who underwent HM1 resection.

Case	Circumferential range of the lesion (%)	Location	Macroscopic type	Invasion depth	Fibrosis	Histology at the positive HM sites	Sites of the positive HM in resected specimen	Adverse events	Additional treatment
1	≥75	Rectum	LST‐G‐M	T1b	F1	Adenoma	Oral, anal	Postoperative stenosis	None
2	≥75	Rectum	LST‐G‐M	T1b	F2	Adenoma	Oral, anal, lateral	–	None
3	≥75	Left	LST‐G‐H	–	F2	Adenoma	Oral, anal	–	None
4	50–74	Left	LST‐G‐M	Tis	F1	Adenoma	Anal	–	None
5	50–74	Left	LST‐G‐M	Tis	F1	Adenoma	Lateral	–	None
6	50–74	Right	LST‐G‐H	Tis	F1	Adenoma	Oral, anal	–	None
7	50–74	Right	LST‐G‐H	Tis	F1	Tub1	Anal	–	None
8	25–49	Left	LST‐G‐M	Tis	F1	Adenoma	Lateral	–	None
9	25–49	Right	LST‐G‐H	Tis	F2	Adenoma	Anal	Intraoperative perforation	None
10	<25	Rectum	LST‐G‐M	Tis	F1	Adenoma	Unknown	–	None
11	<25	Rectum	LST‐G‐M	–	F1	Adenoma	Anal	–	None
12	<25	Right	LST‐NG	T1b	F2	Tub1	Oral	–	Surgery

*Notes*: The right colon includes the cecum, ascending colon, and transverse colon, whereas the left colon includes the descending and sigmoid colon.

Abbreviations: F1, mild fibrosis; F2, severe fibrosis; HM, horizontal margin; HM1, lesions with positive or indeterminate horizontal margins; LST‐G‐H, granular‐type laterally spreading lesion with homogeneous type; LST‐G‐M, granular‐type laterally spreading lesion with nodular mixed type; LST‐NG, nongranular‐type laterally spreading lesion; Tis, mucosal carcinoma; Tub1, well‐differentiated tubular adenocarcinoma; Tub2, moderately differentiated tubular adenocarcinoma; T1b, lesion more than 1000 μm from the muscularis mucosae.

## DISCUSSION

In our study, we found that the characteristics associated with positive HMs were dependent on lesion size, especially lesions with >50% circumference and severe SM fibrosis. Furthermore, the overall rate of positive HM was 6.0%, which was lower than previous reports,^19^, ^20^, ^29^ and no local recurrence was observed in either group. Unlike gastric lesions, colorectal lesions without inflammation of the colonic background mucosa have a thinner mucosa and fewer blood vessels, making it easier to diagnose the lesion boundaries, and thus easier to perform mucosal incisions with less coagulation, which may have contributed to the fewer HM1 events. Positive HM sites in HM1 resections were more frequent at the oral or anal sites than at the lateral sites. The strengths of this study include clarification of the characteristics and sites associated with positive HM resected en bloc with ESD.

Lee et al. showed that the characteristics associated with positive HMs were lesion size, procedure time, and gross morphology.^20^ In particular, there were no significant differences in traction or single balloon‐over tube usage, our study indicated that there was a significantly higher proportion of lesions exceeding 50% of the circumference in the HM1 group than in the HM0 group. This result could be due to the narrower lumen of the large intestine compared to the stomach, and unlike the straight esophagus, the unique flexure and haustra of the large intestine were thought to have affected the HM status. On the other hand, there no difference in the positive HM rate between expert and nonexpert in our study. Not only the small sample size but also the preoperative marking around the lesion were also considered to be the reasons why there was no difference in the HM1 ratio between the experts and nonexperts groups. By confirming the preoperative marking before ESD, the incision was prevented from being close to the lesion, which contributed to there being no difference in the HM1 ratio between both groups. In our study, all 12 cases with HM1 had fibrosis: 4 had severe fibrosis and 8 had mild fibrosis. The prevalence of severe fibrosis was significantly higher in the HM1 group. Fibrosis reportedly leads to lower en bloc resection rates and more complications.^26^, ^30^, ^31^, ^32^, ^33^ Excessive cauterization of the fibrosis near the outer periphery of the lesion may lead to pathological misdiagnosis owing to the elongation of nuclei and creation of an artifact, which contributes to the result of a positive HM.^20^

In long‐term follow‐up in our study, no local recurrence was observed in either group. Previous publications have reported that the 5‐year local recurrence rates of the positive HM cases were 2.1%–40%.^19^, ^20^, ^21^ Moreover, the local recurrence rates of lesions resected with a free resection margin ≥0.1 mm has been reported to be as low as 2.9%.^34^ However, our preoperative markings may have contributed to no local recurrence, which prevented the incision line from being close to the lesion. Moreover, observations of the margin of the ulcer after ESD were important to prevent local recurrence, even in cases where the incision line was close to the lesion or in cases where HM evaluations of resected specimens were difficult due to excessive cauterization.

Previous publications showed characteristics associated with HM1 resections, similar to those in our study^20^, ^21^; however, the histology and sites of positive HM were not investigated. Our study showed that the positive horizontal sites of the resected specimens were more frequent at the anal and oral sites. The reason for this difference in the positive lesion sites may be due to the scope penetrating the fibrotic submucosa, which caused excessive cauterization on the anal side, and the incision line on the oral side was close to the lesion due to its large size, even when preoperative marking was performed before ESD. As a result, the HM status was diagnosed as HM1 if the tumor was present in the initial section of the slices, as in case 1 in the HM1 group.

The current study has several limitations. First, it was retrospective in nature and conducted at a single institution. Second, we were unable to perform multivariate analysis owing to the small number of HM1 cases included. Therefore, future studies should include a larger sample size. Finally, owing to the relatively short follow‐up period, we could not calculate the local recurrence rate over a 5‐year period.

In conclusion, characteristics associated with HM status in en bloc resection with ESD include large lesion size and severe SM fibrosis, but no local recurrence was observed in either group. Furthermore, we suggest that ESD for lesions with these characteristics should be performed with attention to HM status at the oral and anal sites; however, further studies are needed.

## CONFLICT OF INTEREST STATEMENT

Authors declare no conflicts of interest for this article.
